# Stray Current Protection and Monitoring Systems: Characteristic Quantities, Assessment of Performance and Verification

**DOI:** 10.3390/s20226610

**Published:** 2020-11-18

**Authors:** Andrea Mariscotti

**Affiliations:** The Electrical, Electronics and Telecommunication Engineering and Naval Architecture Department (DITEN), University of Genova, 16145 Genova, Italy; andrea.mariscotti@unige.it; Tel.: +39-010-353-2169

**Keywords:** corrosion, impressed potentials, railway transportation, stray current, stray current protection, stray current monitoring, uncertainty

## Abstract

Electrified transportation systems (ETSs) are affected by stray current problems impacting within and outside the right of way on reinforcement, buried metal structures and foundations. Stray current protection systems have recently been integrated in the track structure. Track electrical quantities are, thus, usually measured to assess track insulation and protection efficiency but should be backed up by additional measurements at the affected structures and installations, in order to assess their exposure and risk of corrosion. Ideally, a stray current monitoring system proceeds from the measurement of these quantities, to data collection and archival, to data presentation, analysis and prediction. Feasible sensors and probes, the impact of environmental conditions and uncertainty are considered for the measurement at the physical level. Data analysis is critically reviewed considering the variability of operating conditions and the effectiveness of each quantity as indicator of track insulation and protection efficiency. Given the normal spread of values, for data presentation and interpretation, suitable techniques are considered based on averaging, curve similarity and feature extraction, and also for the task of assessing compliance to limits or reference values and establishing a trend that may drive informed maintenance decision.

## 1. Introduction

Corrosion induced by stray current (SC) is a significant problem of electrified transportation systems (ETSs) and has attracted a lot of attention, especially for systems operated at dc, such as light railways, metros and rapid transit in general: general system analysis [1,2,3,4,5,6,7] including design optimization and defects [8,9], impact on buried conductive pipes [10,11], with both deterministic and stochastic approaches [12,13]. A marginal impact may be expected also for ac railways [14,15], but the lower intensity of the traction current and the lesser impact of ac on the chemical mechanism of corrosion make it a less relevant problem.

There has been a significant modeling and simulation effort in the last 15 years to better understand system dynamics, to identify relevant factors and to optimize design solutions. It is, nevertheless, true that an accurate simulation result stems from a comprehensive description of the system, fed with accurate estimates of all relevant parameters (in particular for rail electrical characteristics, conductance to earth and leakage paths along the fastening system). Low-frequency phenomena can be, in principle, accurately modeled, but there are approximations when the system is quite extended and its electrical parameters are not fully and accurately documented and they may vary with time and environmental conditions: track to earth conductance [16,17,18,19,20] and longitudinal rail resistance (and inductance, for ac railways) [21,22]. Performance of instrumentation under severe environmental conditions is a significant point to consider for instrumental uncertainty, including the characteristics of the electrochemical electrodes used for impressed potential readings at structures. In addition, simulation models, as well as project requirements, need validation by comparison with experimental data [23], obtained with suitable, sound and correct methods; principles are generally known, but the weak points reside in how to manage deviations from assumptions of the few standardized approaches and how to include variability of parameters and their worsening during system life, for which suitable margins should be included.

Compliance to standards and to contractual specifications is generally required, but demonstration methods are rarely clarified. During test and commissioning, track insulation and impressed potential can be demonstrated following the methods in EN 50122-2 [24] and EN 50162 [25] integrated by good engineering practice [19,26,27]. When it comes to electrochemical potentials, great care must be given to installation, preservation of humidity and contamination, which are specific to this field of measurement. An example of non-ideality of measurement methods and of necessary precautions is given in [19,20] for the measurement of track insulation and insulating rail joint efficiency, which are, in principle, well defined and known, covered by standardized methods (EN 50122-2 [24]). Such methods have intrinsic systematic errors, mostly related to the quality of setup earthing (common problem trackside), to the track length included in the setup and the positioning of voltage measurement points.

In addition, extrapolation that accounts for system ageing, adverse environmental conditions and maintenance procedures is not possible unless periodic monitoring is established. This may be implemented as track insulation and impressed potential measurements routinely carried out by a specialized team (accessing the track during night engineering hours) or continuously by a stray current monitoring system (SCMS), operating 24/7. The SCMS features automatic logging, can remove human error (e.g., during measurements and when collecting data, especially if short time is available) and is economically more efficient (if not only the initial cost but also savings in man-hours during system life are included). It can also give indications for preventive maintenance and corrective actions.

In general, the quantities that an SCMS should measure and analyze (its “physical scope”) do not have full normative references, are not univocally identified (although there is common understanding among experts) and, in general, are not extensively covered in the available scientific literature [26,27,28]. Similarly, what the SCMS can measure should be what can also be measured and verified by the engineering team. In addition, performance and characteristics of SCMS software for analysis and prediction are poorly documented in terms of used algorithms, sensitivity and detectability, and also human–machine interface capabilities (what we may call the SCMS “functional scope”). There are some SCMS “products”, developed by major companies of the electrified railway sector, which are not extensively described nor characterized in the accessible documentation, in particular for the following important aspects:

- Number and type of measured quantities of track, exposed structures and substations;
- Ability to discriminate traffic patterns, such as peak hours, off-peak hours and weekends;
- Implementation of trend and criteria used for medium- and long-term analysis;
- Interaction with and presentation to the user (human–machine interface, HMI) and guided decision criteria;
- Performance and effectiveness and how they can be verified and demonstrated.

Whereas voltage and current measurements at the track, in principle, share the same accuracy as other low-frequency electrical measurements carried out in an industrial context, impressed potentials, concrete resistance and electrical insulation readings are intrinsically less accurate. The insulation conditions, especially for exposed parts, are heavily affected by the moisture percentage and condensation, which may occur quickly due to environmental changes and may differ from day to day, and also some variability between morning, afternoon and evening cannot be excluded, depending on night condensation and the amount of received sunlight (insolation).

In addition, the ETS operating conditions are not perfectly repeatable, in that driving style, unpredictable small delays and number of passengers all influence the acceleration and braking profiles, their intensity and the amount of absorbed or released traction current.

This work, thus, aims at reviewing the electrical quantities that characterize stray current, stray current protection and corrosion phenomena and are suitable for SCMS monitoring, identifying both their typical behavior and their usefulness (alone or combined) as indicators of the health conditions of the system. First, a typical system operation and the criteria for the stray current protection system (SCPS) sizing are discussed. Second, measurement of the relevant quantities is considered, discussing typical sensors and measurement setups and their characteristics. SCMS desirable capabilities are then considered, focusing on the best electrical quantities in terms of representativeness of system insulation health status. Last, methods to verify the accuracy and effectiveness of an existing SMCS are proposed using simulated current leakage scenarios.

## 2. Stray Current Protection

The subject of stray current protection (SCP) is considered, together with a description of the operating scenarios, in order to identify the relevant quantities, constraints and desired outputs.

For the phenomena of stray current and corrosion, the relevant ETS parts are the track and structural elements nearby (e.g., viaduct deck, tunnel lining and track-supporting structure), metal works and platform. Such systems are typical metro or rapid transit systems featuring tunnels or viaduct sections, platform screen doors, visible cable trays and walkways and items of electrical equipment located wayside.

**Figure 1:**
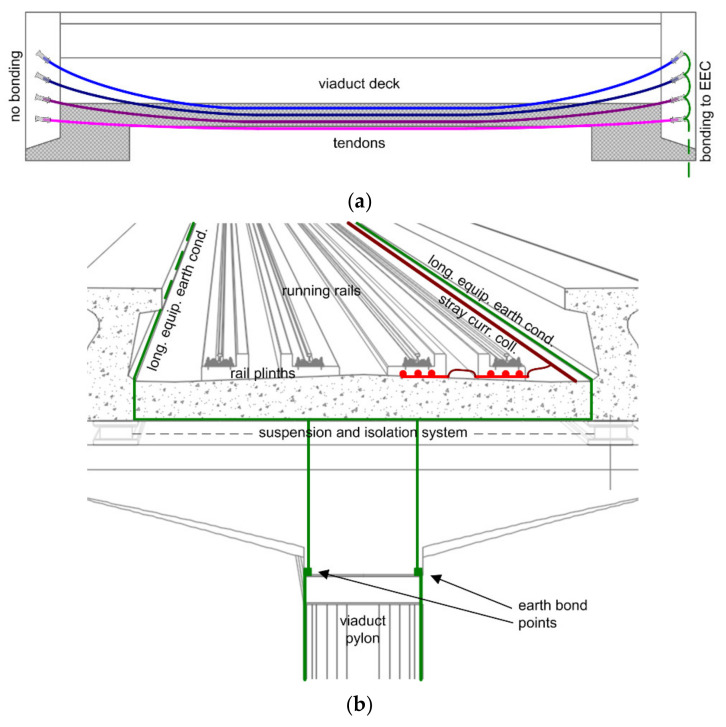
Details of (**a**) a viaduct span with tendons (earthed at one end only), (**b**) a viaduct deck and pylon with sketch of the earthing system (in dark green) and of the stray current protection system (SCPS) (in red for stray current drainage (SCD) elements, in brown for the bonding connection and stray current collector (SCC)).

The attention is focused on several structural elements that might be exposed to corrosion, partly or fully, caused by stray current and whose construction characteristics must be known in order to devise suitable measurement arrangements. The main elements that follow are then sketched in Figure 1.

- Track and track bed. Rails are laid down in a wide range of arrangements, with sleepers or continuous support, the former divided into mono-block and twin-block sleepers; all these types of track bed are built bottom-up, starting from a compacted supporting layer whose name differs between railway and metro applications at grade and tunnel—usually named hydraulic bonded layer at grade and filler layer inside tunnels (where, depending on tunnel lining, it also accomplishes the task of anchoring to lining voids). A concrete-bearing (or supporting) layer is built with various amounts of reinforcement and concrete filler at edges and around sleepers, including bituminous insulation of the latter. An alternative way of track construction is top to bottom, using a pre-assembled track, where sleepers are replaced by concrete mono-blocks, each provided with a supporting pad and plastic/rubber boot—this arrangement is put in place suspended over a pre-constructed reinforcement layer and concrete pouring completes the installation. Before pouring, stray current drainage (SCD) elements may be inserted, with the most practical solution being an SCD mesh that horizontally covers the track area up to the blocks profiles.
- Reinforcement is provided as supporting layer for the track bed, as well as at many other locations around the track bed and at tunnel walls and the viaduct deck. Besides providing mechanical stiffness all along the reinforcement cage and where rebars overlap, there is no convenience in a solid metal–metal connection, which would otherwise drastically reduce the longitudinal electrical resistance, attracting, as a consequence, stray current (see Section 2.1.1). The reinforcement design will also host the SCD rebars put in place and secured by means of spacers, which ensure the minimum clearance from construction reinforcement and that the distance from the rail foot is conveniently small (see Section 2.1.1).
- Tunnel sections. Construction technology may be divided into tunnel lining with prefabricated panels (usually six or eight along the circumference of the tunnel) or continuous concrete casting (concrete spray) after the tunnel boring machine, together with the inclusion of additional structural frames and rock bolts as supporting structures. A completely different technique is that of cut and cover, suitable for shallow tunnels and for junction section between deep tunnels and at grade; a rectangular section is excavated and supported by lateral walls and roofed with an overhead supporting structure, all built with standard reinforced concrete and, thus, potentially exposed to stray current. Longitudinal electrical continuity should be broken by isolating diaphragms.
- Viaduct span is usually insulated, if propped up on pylons and an abutment, by a polymeric suspension system that absorbs vibrations and allows some movement of parts; electrical continuity for electrical safety and lightning protection is provided through a longitudinal earth equipotential conductor (EEC) that is in, general, bonded together with discharging rebars inside the pylons. Although suspension elements are not thoroughly disciplined by international standards, local authorities have issued regulations to ensure high levels of electrical insulation to test 100% after installation and viaduct construction is finished. Usually, the required insulation levels for a new system are in the range of 10–100 MΩ.
- Pylons and foundation plinths, besides being structural elements, are dispersing electrodes, useful to implement distributed earthing and ensure a sufficiently low resistance to earth for installations at viaducts, especially if providing earthing to a lightning protection system. To this end, some rebars are specifically welded together and form a low-resistance circuit, electrically accessible for connection on top and dispersing at the base of the pylon (where deep soil with lower resistivity is easily reached). The typical arrangement of such an earthing circuit is the parallel connection of 10 to 20 vertical rebars, equipotentialized by horizontal bonding by means of smaller circumferential rebars every 1 m or so in height or using a spiral rod from bottom to top. All elements that are part of such earthing circuit must be welded. Pylon potential is accessible at earth plates, usually provided on top and at the ground level of each pylon.
- Tendons are a longitudinal structural element that may accidentally provide an electrically continuous path for stray current between viaduct spans; tendons’ heads should be completely insulated, because otherwise, there would be a concentration of any picked up stray current. However, as conductive parts, they must be earthed, and they are usually earthed with a single point bonding to the longitudinal EEC. Due to unavoidable mechanical stress, some influence of environmental agents and, possibly, human errors during construction, tendons are quite exposed to the risk of stray current coupling and must be checked for initial electrical insulation during commissioning and might be included among the elements to monitor.

The amount of track leakage current and the way it is distributed at the interface between rails and infrastructure highly depends on the construction technique, the interfacing elements and suitable isolation techniques, such as non-conductive pads and bituminous layers, as concisely examined earlier in this section. Considering the pros and cons of each construction method and the precautions to improve the effectiveness of leakage prevention is beyond the scope of this work. From a sensing and measurement perspective, there is no remarkable difference as the “accessible terminals” for the evaluation of stray current quantities remain unchanged, both for the track itself and, possibly, coupled structures along the alignment.

The degree of exposure of wayside structures may increase with time and depends, most of all, on the ageing of structural insulation (implemented with polymeric or concrete layers); concrete carbonation, worn out points due to mechanical stress or initial unnoticed construction defects or, to a much lesser extent, improper maintenance all cause water ingress and consequential trapping at interfaces. For this reason, selected structures should be monitored by the SCMS, especially in the presence of other significant external sources, such as photovoltaic plants or other transportation systems (at exchange stations and crossings), because monitoring the track alone would not exhaust the list of possible sources of stray current.

Considering the source of stray current (SC), the circuit is composed of the track with its mechanical fastening to the track bed (or sleepers), backed up by the stray current protection system (SCPS), introduced in track construction in the last 20–25 years.

### 2.1. Stray Current Protection System

SCPSs have been devised in modern projects to intercept part of the leakage current that leaves rails, capturing it through stray current drainage (SCD) sections and collecting it into a wayside stray current collector (SCC), back to the traction power station (TPS).

#### 2.1.1. Stray Current Drainage (SCD)

**Figure 2:**
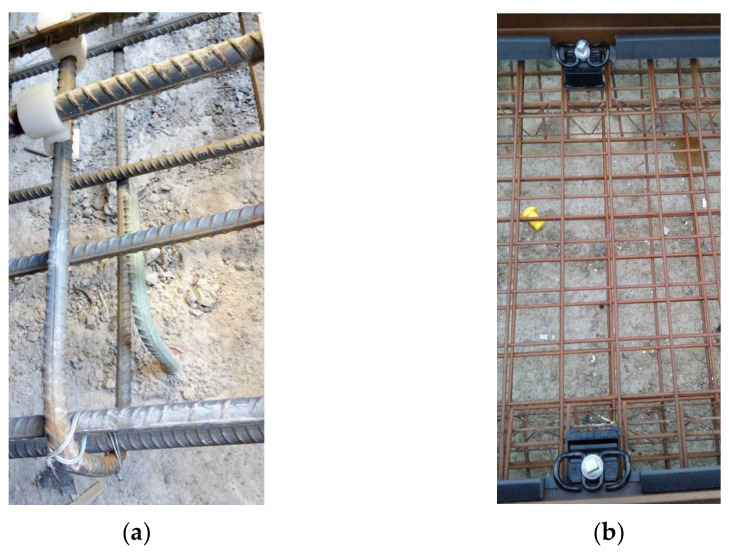
Details of stray current drainage using (**a**) rebars and (**b**) grids.

SCD is implemented by means of specific independent longitudinal rebars or a reinforcement grid, located beneath the running rails and intercepting the stray current leaving the rails along their length (see Figure 2). An important point is that these elements must be insulated from the remaining reinforcement of the track bed and, at the same time, have a good electrical interface with the leaking elements of the track, maximizing the conductive coupling with them. For structural reasons, putting insulating sheets beneath them is out of discussion, as they would weaken the structure, especially considering the intense lateral forces. So, at present, insulation relies upon different cement thicknesses above and below the SCD elements.

Current designs use an amount of capturing steel between 600 and 1000 mm^2^ total cross-section for one rail; it is noted that the total cross-section for UIC60 rails (60 kg/m and equivalent) is 7660 mm^2^. Construction steel usually has an electrical resistivity that is 13–14 times higher than copper. As mechanical properties of these capturing rebars are of secondary importance, selecting steel with lower electrical resistivity may be an option; a candidate would be low-grade carbon steel (SAE/AISI classification 10xx) that features ratios of 8.7 to 10.7, increasing the effect of the installed cross-section by about 50%. However, resistance to corrosion is poor and tests with various chloride concentrations in concrete have shown that the expected life is in the order of 10 years, even for moderate chloride concentrations [29].

When using rebars, they are usually distributed evenly beneath the rail foot; a wider spread of such rebars must take into account interference with the vertical booths housing the screws holding the fasteners. Similarly, when using a capturing grid, booths must be made to correspond to the centers of grid meshes. The separation between the SCD rebars/grid and the rail with its fastening system should be minimized in order to maximize this transversal conductance term and maximize capture efficiency. Ideally, SCD sections should capture the leaking current and confine it until the SCC is reached. However, this will work alongside a symmetrical mechanism of further leakage from the SCD circuit to the rest of the reinforcement. Various solutions may be conceived and they are not mutually exclusive: (i) selective increase in the resistance of the transversal current path by means of an insulating layer, provided that mechanical characteristics are preserved; (ii) use of a thicker concrete layer in the lower part than on top; and (iii) use of the lowest attainable values of SCD longitudinal resistance, contrasting the leakage path through transversal resistance. The third point is the most easily implemented and is what is normally done, supported by the second solution, where the concrete layer supporting such capturing rebars and separating from the other parallel reinforcement rebars is usually 2–3 times thicker than that beneath rails. The insulating layer indicated at the first point has never been seen to be used for structural reasons, especially considering lateral and tilting forces.

It is, however, understood that the presence of the SCD at such a short distance slightly worsens the insulation of the running rails, which would be higher if the SCD elements were removed, replacing them with concrete, leaving the rest of the geometry unaltered [8].

**Figure 3:**
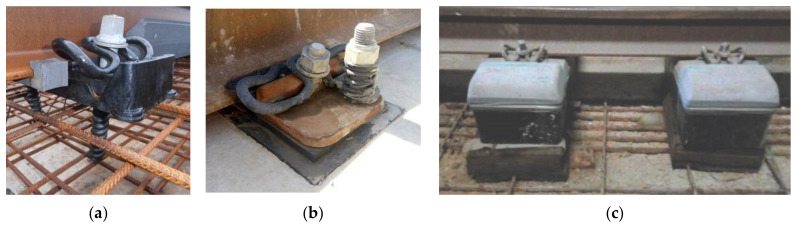
Details of (**a**) a fastener booth and insulating block, (**b**) insulating pads and (**c**) the floating blocks technique.

Leakage current may also leave the rail as surface leakage over the insulating pads (in the case, e.g., of excess moisture and dirt) and then beneath them onto the concrete surface or right along fasteners and screws, in the case e.g., of a damaged booth, which exposes the screw to a conductive path to the surrounding concrete. In this latter case, the effectiveness of SCD to capture the resulting leakage current around the booth is lower, as the leakage path is vertical and quickly moves away from the protected area beneath the rails. Booths may be damaged during concrete casting or as a consequence of tightening of an incorrectly positioned screw. Although one may think that an open booth at the bottom may avoid humidity stagnation, cement may also ascend due to the high casting pressure; the best solution is a sealed booth filled with special grease that avoids water and humidity ingress. Conversely, the technique of floating blocks and a pre-assembled track is quite immune to this kind of issue because the fastening system is pre-assembled and well separated from the parts interacting with poured concrete, and current leakage occurs mostly along the block surface. Some details are shown in Figure 3.

There is no definite reliable rule to estimate the necessary cross-section of SCD rebars; the intention is maximizing the amount of *I_scd_* current diverted from the stray leakage path that would otherwise go through the track bed. A first factor is the shape of conductors and the equivalent cross-section transversal to the expected leakage current path; larger SCD rebars will more easily capture leakage current flux, but also a larger number of smaller ones, which have the advantage of some mm less in thickness and, thus, are better insulated from the rest of the reinforcement (as explained later). Then, the comparison of the resistances of the two current paths (through the SCDs back to the SCC and/or TPS, and through the rest of the concrete, reinforcement and to earth) accounts for such effectiveness and the target *R_scd_* value. An equivalent resistance of reinforcement and concrete may be estimated as follows.

- The flow of current occurs both longitudinally and vertically towards the rest of the structure and the soil beneath. Depending on the construction details, the longitudinal path may be significant or not; if the upper part of the track bed is arranged in sections of limited length with gaps (often called “plinths” or “rail plinths”), then the longitudinal continuity is broken by such gaps. Similarly, if the track bed reinforcement is designed with non-touching reinforcement that is, thus, structurally, but not electrically, continuous, the longitudinal conduction is also not an issue; this occurs, for example, if gaps or buffer diaphragms are provided for mechanical movement. In these two cases, the contribution of reinforcement to longitudinal conduction is reduced, not to say negligible. It is also advisable that construction reinforcement is realized with coated rebars (yet more expensive), oppositely to the naked rebars used for SCD. These plinths and a shallow track-bed mat are then anchored to the underlying viaduct or tunnel floor with C-shaped rebars (often named “shear connectors”), and this is the second vertical conduction path.
- For a track bed of width *w* and height *h*, the longitudinal resistance per unit length *r_bed_* may be calculated assuming uniform concrete: *r_bed_* = ρ*_c_*/*wh*; for a *w* = 5 m × *h* = 1 m construction *r_bed_* amounts to 10–40 Ω/m for ρ*_c_* ranging between 50 and 200 Ω/m. It is evident that uncoated reinforcement prevails by the effect of its lower resistivity, despite the low filling percentage *k_f_* (using, e.g., one T20 rebar every 15 cm, then *k_f_* = 0.014). Steel resistivity amounts to a fraction of μΩm, but the tiny layer of concrete between overlapped (but not touching) rebars must be considered to estimate the overall electrical continuity; this layer adds, in series one, to some Ω, every few m (where rebars overlap). This occurs for several tens of rebars in electric parallel and for more than one mating point per rebar (including crossings), thus, overall, accounting for *r_bed_* in the order of 0.1 Ω every 100 m, or 1 mΩ/m (confirmed by site measurements in Copenhagen Metro). If coated rebars are used, then the concrete resistivity only remains larger by several orders of magnitude.
- The overall track bed may be efficiently shunted by the SCD rebars if they have a longitudinal resistance that is a fraction of *r_bed_* by about 10 to 20%, leading to an advisable p.u.l. resistance *r_scd_* of 0.1–0.2 mΩ/m.

When considering SCD sections, it is evident that the short connecting cables at the extremities are of less importance and moderate cross sections of Cu conductor may be used, favoring flexibility and widening the range of suitable clamp-on probes (probes for some tens of A range do not feature a large bore diameter). Considering a reference 200-m long SCD section, a 35-mm^2^ Cu conductor of 5-m length would add 6% resistance to a 200-m SCD section made of 6 × 16 mm diameter steel rebars (*R_scd_*__200m_ = 40 mΩ); increasing it to 50 mm^2^ brings the added resistance to only 4.3%. The corresponding longitudinal track resistance for 200 m is about 3.5 mΩ (UIC60 or similar sections, including welding points [21]). For the additional SCC series resistance, please see Section 2.1.2.

#### 2.1.2. Stray Current Collection

The captured stray current in each SCD section needs to be brought back to the TPS negative, and this is usually accomplished by means of a stray current collector (SCC), although there are solutions where the SCC is not installed and the SCD sections are daisy chained (implementing the function of drainage and collection altogether). This latter approach has two disadvantages: the longitudinal resistance of the collecting means is larger, since it is that of the daisy-chained SCD sections themselves; the collected stray current is in contact with the concrete of the rail plinths all along its way back to the TPS, so additional leakage (or a lower efficiency) must be considered.

A hybrid approach may also be used, where the SCD sections are daisy chained but backed up by the external SCC, which reduces the overall longitudinal resistance and de facto keeps the drained current simply thanks to the significant difference in longitudinal resistance compared to the SCD sections.

The SCC is simply an insulated conductor guaranteeing that the stray current, once collected by each SCD section, does not further leak into the system and is brought back to the TPS negative with the lowest longitudinal resistance attainable, compatible with cost and space constraints and taking into the due consideration that the overall SCPS efficiency marginally increases when a certain cross-section value S_scc_^*^ is reached.

SCC sizing may be achieved by considering that its longitudinal resistance is in series with the SCD sections, so their performance is slightly reduced. A first approach is to compare the SCC p.u.l. linear resistance *r_scc_* with that of the SCD (*r_scd_*). The SCC cross-section threshold value S_scc_^*^ is, thus, a fraction of the SCD cross-section *S_scd_*, following the ratio *q* of the respective resistivity values (which, for copper and construction steel, is about 13) and keeping a convenient oversize factor.

A 120-mm^2^ Cu SCC (commonly considered as a convenient cross-section for the longitudinal equipotential earth conductor, EEC) has a resistance of 2.8 mΩ every 200 m (the assumed SCD length), slightly less than that of the track section (about 3.5 mΩ). The previously calculated *R_scd_*__200m_ of 40 mΩ indicates that the impact on the SCD efficiency of the SCC resistance for a similar length is quite limited (7% in this case). It appears, however, that the farther the SCD sections are from the TPS, the longer the SCC resistance is in series before the final collecting point at the TPS; for *L_TPS_* = 3 km of TPS-to-TPS separation, at the track mid-point, the SCD will see an SCC resistance that is the parallel of two *L*/2 sections, 3.5 times larger than the resistance seen by the second SCD section at only 200 m away from the TPS (with the first SCD section in front of the TPS seeing 0 length and 0 resistance). The overall efficiency would, thus, be lower, right where the expected stray current is maximal, when the train is pulling current around the mid-point (and the track voltage is maximal); additional series resistance of 24.5% would reduce the efficiency to 80% of the original one for the SCD alone (simply obtained as 100%/(100% + 24.5%)). For this reason, the SCC should be sized taking into account the track mid-point; taking a 240-mm^2^ SCC (that halves the initial SCC resistance), the worst-case efficiency reduction would be as high as 89%. As a matter of fact, typically selected cross-sections for the SCC are larger than those of EEC, namely in the range of 240–300 mm^2^.

These figures are in agreement with those appearing in [30], simulated for a system with a very low soil resistivity (10 Ωm) and a limited thickness of the supporting structure (about 0.3 m), that hosts the stray current mat in contact with the soil beneath; the efficiency increases more significantly up to about 360 mm^2^ of SCC cross-section but the overall efficiency values are slightly lower because of the very low soil resistivity used in the simulations. In reality, the track bed is separated from the soil in every part of the alignment; at viaducts, this is quite intuitive, and at grade, the supporting civil structure is more than 1 m thick and laterally extended before compacted soil is reached. Inside tunnels, tunnel lining is circumferential, thus wrapping and separating the track bed from the surrounding soil. As all such concrete and construction layers are also treated for water tightness and protection against natural corrosion (e.g., by insertion of drainage and isolating layers), the track bed and the SCPS can be considered buffered from the surrounding soil by a medium with an equivalent resistivity of at least that of dried foundation concrete (90–100 Ωm). The conclusion in [29] is confirmed, that in low resistivity soils, the SCPS efficiency may be very low, even for very large amounts of installed SCPS steel and copper; in this case, buffering by intermediate structures and layers is even more important and should be considered.

#### 2.1.3. Overall SCP Efficiency

We have seen that the local efficiency calculated on each SCD section must be corrected for the longitudinal resistance added by the SCC (similarly, when SCDs are simply daisy chained, also implementing, in this way, the collection function).

In addition, a second determinant factor is the environmental temperature causing the increase in the longitudinal resistance values; with estimates carried out at a room temperature of 20 °C, an air temperature of 50 °C and a surface temperature of 70 °C, elements installed in the track area under the sun cause a further reduction in the SCP efficiency. The temperature coefficient of the resistivity of copper and reinforcement steel is about 0.4 and 0.3%/°C, respectively, so the overall increase of 50 °C in temperature amounts to 15–20% [21,22]. Assuming that the transversal conductance through the concrete does not change significantly with temperature, efficiency will reduce by the same amount.

It is, thus, important to size the SCP elements for optimized efficiency at the operating points of the system, for pulling current midway between TPSs and at a conveniently high temperature. Experimental verification by means of direct measurement is, thus, important for the verification of such variability and its impact on the stray current budget.

The longitudinal resistance and the transversal conductance of SCP elements may be measured with standard methods. A 4-wire Kelvin setup is advisable for the longitudinal resistance of each SCD section and of the joining cable, including contact resistance at joints. Once disconnected, the transversal conductance should be measured with a volt-amperometric method using a voltage source (such as a battery), because insulation meters cannot feed the necessary current intensity, being the equivalent insulating resistance in the range of some Ω or tens of Ω. The obtained values allow verifying compliance to design parameters and having a first estimate of the SCP efficiency.

**Figure 4:**
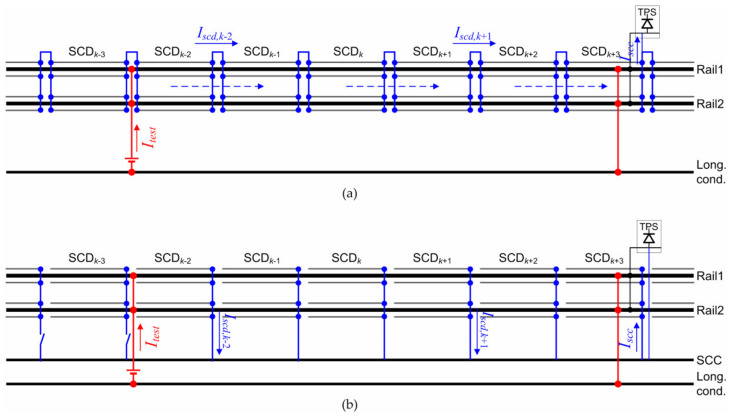
Typical SCPS arrangements and measurement of SCP efficiency: (**a**) SCPS without SCC and daisy-chained SCDs; (**b**) SCPS with SCC and isolated SCDs. SCPS elements are in blue; the test circuit is in red.

The overall SCP efficiency can be measured with the following setup (see Figure 4). A test current is applied to a track section, e.g., by means of a battery system, whose return is done through an independent conductor, such as the longitudinal EEC. This replaces the current pulled by running trains with a stable and known source, whose output current is known. In order to have a similar distribution of potential, the test section is terminated in front of the TPS, where the SCPS is also terminated onto the TPS negative. Then, the *I_scd_* and *I_scc_* quantities can be measured along the tested section by dc current clamps.

The efficiency of the single SCD section *k*, *E_scd_*_,*k*_, is given by the ratio of the drained current *I_scd_*_,*k*_ and total stray current *I_tl_*_,*k*_ in that section [26,31], *I_tl_*_,*k*_ = *I_tr_*_,*k*+1_ − *I_tr_*_,*k*_. The overall efficiency *E_scp_* may be considered as the average of all SCD sections or as the ratio of the SCC current at some point, such as midway between TPS and the total stray current (available straightforwardly from a model [1], Sec. III.B, but recoverable from track current measurements midway and at TPS in a real system).

(1) $$E_{scd,k}=I_{scd,k}/I_{tl,k}\;\;\;\;E_{scp}=\frac{1}{K}\sum_{k}E_{scd,k}$$

Provided that the train return current is known with sufficient accuracy, the SCP efficiency can be measured in real time by the SCMS, if the SCP currents are included among the SCMS quantities to measure.

#### 2.1.4. SCP Operation

There are two options for the SCP operating condition: the SCP may be “switched on,” only in case significant track leakage is found, or it can be operated continuously as a stray current reduction method. The switch on is achieved by closing the links of the SCD sections and by connecting back to the TPS negative.

As for the track connection to the TPS, different circuit arrangements have been analyzed in the literature: the connection is direct or established through a diode or a controlled switch (such as a thyristor, or two thyristors in anti-parallel, serving both polarities) [2]. As a matter of fact, the track and traction system design has oriented to a floating track, preferable to a TPS grounded track, but also to more complicated schemes involving diodes, thyristors and switches, also because they were not exempted from situations of large track voltage and hot spots. In any case, electrical safety must be ensured by the use of voltage limiting devices (VLDs), also named overvoltage protecting devices (OVPDs) [32].

A completely floating track has its potential to earth distributed all over its length, from a negative value at TPS to a positive value under the train, assumed at some distance away [30]; a grounded TPS negative imposes a zero potential onto the connected track and, thus, the potential to earth at the train doubles, increasing, as a matter of fact, the amount of stray current. The maximum stray current calculated with a single end-fed line with train at distance *L* indicates a ratio of four of stray current between the two situations of floating and grounded track [30]—the reason is not only the doubled maximum track voltage but also the doubled length for the grounded system where such maximum occurs (at the end, not in the middle, with the TPS grounding at the other end).

(2) $$I_{tl,\max,\mathrm{FLT}}=\frac{IR_{tr}L^{2}}{8R_{te}}\;\;\;\;I_{tl,\max,\mathrm{GND}}=\frac{IR_{tr}L^{2}}{2R_{te}}$$

where *R_tr_* and *R_te_* are the track and track-to-earth resistance, *I* is the pulled current and *L* is the train position as distance from the reference TPS (at 0 m).

Of course, these are the maximum values of stray current under an ideal and a pessimistic arrangement of single-end fed track.

### 2.2. Track Potential and Stray Current

The relationship between the track potential distribution and resulting stray current is very important for the use of the former as an indicator of track insulation and stray current intensity, as commonly done by SCMSs. The track potential is influenced by the operating conditions of trains along the line, and values should be always considered with respect to the local earth potential; negative values, for example, may occur, depending on the track termination at TPS.

The distribution of the track potential is, thus, not intuitive and several factors concur to its waveshape vs. time and longitudinal position (the chainage):

- Traction and braking phases alternate, interspersed with coasting and cruising phases;
- Rails and tracks are bonded along the way (rail-to-rail and track-to-track bonding), achieving the maximum track balance and allowing a wide exchange of regenerative braking current towards tractioning units on the same or different tracks;
- TPSs are distributed along the alignment in an approximately regular pattern and situations of single-end fed trains do not occur (exceptions are temporary TPSs out of service and some portions of depots and yards, where trains, however, travel at very low speeds, with low traction effort and absorbed current).

**Figure 5:**
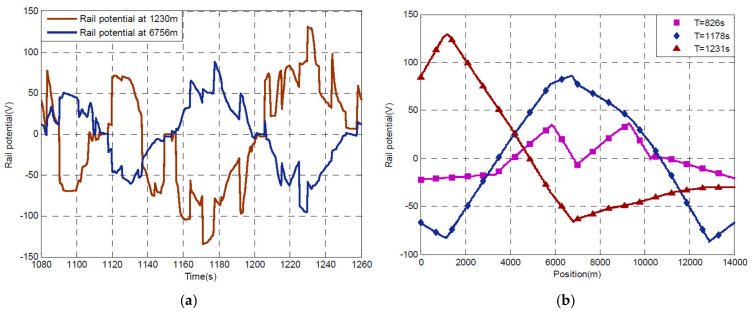
Example of track voltage variability vs. time (**a**) and position along the track (**b**). Reproduced from [7].

**Figure 6:**
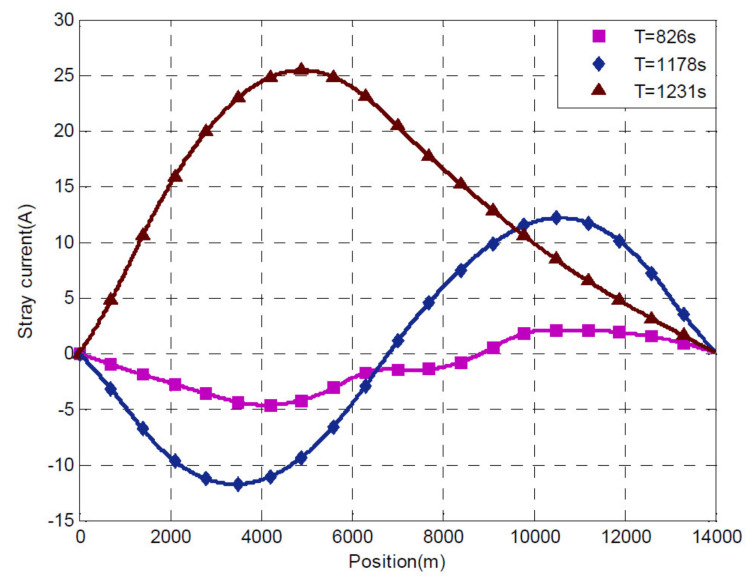
Example of stray current vs. position along the track. Reproduced from [7].

Finding a proportionality or curve similarity between the track voltage *V_te_* and the resulting stray current is particularly difficult, as shown in Figure 5 and Figure 6.

It is easy to see that the excursion of the track voltage is significant at any given track longitudinal position with ±100 V and larger for the system considered in [7] (see Figure 5). This is confirmed by vertically sampling the three curves in Figure 5b that refer to position 6756 m, whose time curve is the blue curve in Figure 5a.

The non-intuitive relationship between the track voltage profile and the stray current intensity is also confirmed by the stray current calculated for the same simulated system [7], shown in Figure 6. At the same longitudinal position of 6756 m, a slightly negative track voltage (purple curve at 826 s in Figure 5b, about −5 V) and a significantly positive track voltage (blue curve at 1178 s in Figure 5b, about +80 V) produce almost the same stray current values of about −2 A and +2 A, respectively, as shown in Figure 6; the disproportion is by more than an order of magnitude.

### 2.3. Electrical Quantities of Interest

Considering applicable standards [24,25,32,33,34] and typical site acceptance tests (also addressing specific local regulations), the quantities to measure for stray current assessment are as follows.

- Track potential *V_te_* with respect to earth is advised in EN 50122-2, App. B, as an indication of track insulation without a clear indication of criteria, besides instructing to extract the minimum, the average and the maximum of the collected *V_te_* samples over a significant time interval. A suitable reference value might be the 5 V threshold of the EN 50122-2 itself, used to specify the track-to-earth conductance limit starting from a current limit of 2.5 mA/m.
- Potential shift of reinforcement in concrete accessible by means of on-site temporary probes or embedded electrodes. Suitable limits are 200 mV for steel in concrete (EN 50162 [25]), including the entire scale with 200 and 350 mV potential shifts, discussed in more detail in Section 3.1.4.
- Potential of accessible conductive parts *V_ce_* with respect to earth is an indication of chance of corrosion, e.g., at the junction point with concrete foundation or fastener to concrete wall (examples are the supports of handrails, walkways, cable trays, etc.), as well as cases of steel in soil (examples are pipes and tanks). Suitable limits may be identified as above for impressed potential shift and for steel in soil between about 100 and 300 mV for steel in soil with variable soil resistivity [25].
- Longitudinal voltage drop *V_cl_* on conductive parts, such as reinforcement, is another indicator of chance of corrosion, as per Annex C.2 of EN 50122-2 [24], more suitable for calculation than for site measurement and monitoring.
- Current leakage from a track section *I_tl_*, drainage current *I_scd_* or collected current *I_scc_* flowing in the SCP at longitudinal position *x*. The accessible SCP quantities *I_scd_* and *I_scc_* indicate the amount of captured stray current and, thus, may be used to indirectly determine the amount of track leakage and of stray current flowing into and outside the track bed. It allows indirect monitoring of track insulation and the assessment of the SCP system efficiency [26,30].
- Track insulation resistance (vs. earth) is usually covered by specific tests during commissioning and acceptance phases [19,20,24]; it could be estimated on-line if, besides track potential *V_te_*, track leakage current *I_tl_* is also available (we will see that the estimate of *I_tl_* from the measured track current *I_tr_* is quite difficult). An alternative method suitable for tramlines and other at grade lines with minimal infrastructure is the one in Sec. A.4 of EN 50122-2 for “track sections without civil structure”, using the information of the gradient of the electric field in the soil and, thus, necessitating two measuring electrodes driven in the soil at two different orthogonal distances from the track, such as 1 m and 10 to 30 m, respectively.

## 3. Stray Current Monitoring System

In principle, an SCMS should collect all the relevant quantities discussed in the previous section in order to offer a representative and comprehensive picture of the system conditions of the stray current protection level and of its evolution with time. Of course, an opposite requisite is economizing on the number of measurement channels and probes and, as a consequence, installation and maintenance. Another element that influences the implementation is that the lack of standardized requirements and technical references makes the contractual specifications vague and weak, reduced to a simple “there must be a stray current monitoring system to check stray current protection”; there is no quantitative specification for the relevant aspects discussed in the Introduction and there are no benchmarks and no acceptance criteria.

Since there are no examples of SCMSs whose characteristics have been discussed in accessible literature, we analyze, in the following, desirable performances for the measurement of electrical quantities (what we may call the low-level physical scope) and for data synthesis, representation and analysis (the part interacting with the user, adding value to the measured data). Finally, in Section 3.3, the variability of operating conditions (including traffic, passenger load and deviations from schedule), environmental conditions and external stray current sources are considered as sources of uncertainty.

### 3.1. SCMS Physical Scope: Measurement of Electrical Quantities

The widest SCMS physical scope may be defined based on the discussion of Section 2.3, where all relevant electrical quantities available tracksides have been considered. Desirable characteristics for the measurements of electrical quantities may be derived by instrumentation commonly used for site tests of track insulation and impressed potentials [26]. Measurement of the stray current flow along the SCPS parts and estimation of the stray current protection efficiency are not required by standard, but they are discussed in some publications [26,27,28,30] and are used as the starting point to identify suitable measurement techniques, besides good engineering.

#### 3.1.1. Track Voltage Measurement

As long as only the track voltage is acquired (as is done by many SCMS realizations), this is not a critical measurement, in that large values are expected (no issues of sensitivity and resolution) and the earth reference may be, in extreme cases, shared between nearby locations (although galvanic isolation is preferable). The measurement is usually implemented at voltage limiting devices (VLDs), that may be deployed at TPSs or, in some cases, at all stations. When only TPSs are equipped with track voltage measurement points, not only are such points quite sparse (separated, e.g., by 2–4 km) but readings are taken in front of the TPS, where the track voltage change is the least (the track resistance from the power supply point is minimal), with an arguable representativeness and comprehensiveness of such track voltage monitoring. This is also confirmed by [4], Figure 3, where the maximum stray current (at train position) is minimal when the train passes in front of the TPS. When VLDs are located at all stations (e.g., for electrical safety reasons), then the density of the measurement points is increased and track voltage is monitored about every 0.6–1.2 km.

A much better, but more expensive, solution would be providing track voltage measurements at every SCP junction box, where current measurements can also be carried out (see below).

#### 3.1.2. SCP Current Measurement

Returning to SCPS efficiency definition and aiming at monitoring its operation, SCPS current measuring points at SCD and SCC should be added. In addition, we saw that *I_scd_* is a good estimate of the track leakage *I_tl_*. A full coverage of the track would imply a measurement at every drainage section, usually designed with a span between 100 and 300 m. For cost saving, not all drainage boxes would be equipped with current-measuring devices.

Suitable approaches to current measurement are represented by shunts and Hall-effect probes. Since galvanic insulation is a necessity, in order to avoid transfer of potential and allow interconnection of remote points, their costs are quite similar (with the shunt resistor to be interfaced by means of an isolation shunt-sense amplifier). Assuming the worst-case leakage of 2.5 mA/m, the drainage sections would capture, when switched on, at most, between 250 and 750 mA, depending on length. However, with the protection in case of a detected local loss of track insulation being the main purpose of the SCPS, a larger current may be occasionally expected as the result of an extreme deterioration of the insulation within a section. At the lower end, the sensitivity of the measuring channel should be able to discriminate a very well-insulated track (e.g., up to 1 kΩ/km); assuming, as reference, a low track voltage of 1 V, the resulting current is 1 µA/m, so a total of 0.1–0.3 mA over the said SCD sections, which represents a lower bound to the desirable sensitivity.

Suitability and performance of current sensors available on the market must be assessed from two viewpoints: (i) robustness with respect to the environmental conditions for a trackside device; (ii) metrological performance, in terms of accuracy, uncertainty and non-linearity, including, in particular, the effect of temperature excursion (that is also significant in the short term, when passing from shadow early in the morning to direct sunlight at mid-day). Taking, for example, two different current measurement methods, the various aspects are quantitatively discussed below: a current probe manufactured by LEM for trackside applications (mod. PCM 10-P [35]) and a custom designed shunt resistor buffered by an isolation amplifier with similar characteristics; the two are indicated as “PR” and “SH”, respectively.

The PCM 10-P has a 4–20 mA output mapped on a ±20 A reading range; this current must be read with a loading resistor of 50 to 250 Ω to remain in the probe capability. Shunt resistors of a suitable rating range from some to some tens of mΩ and 2 to 5 W of power rating [36]; the output of the shunt must be optimized to feed the isolation amplifier, with a large enough signal to improve the signal-to-noise ratio. As an example, the AMC1302 [37] will fit a 50 mΩ shunt for a 1 A full-scale current (SCD) and a 5 mΩ shunt for the SCC current (supposed, at most 10, times larger). The characteristics of the two devices with respect to environmental conditions and metrological performance are discussed in the following and then summarized in Table 1.

1. Environmental conditions:
  - Vibration and shock. The wayside environment is quite exposed to vibrations and shocks, caused by the interaction of the rolling stock with the track, including the effect of rail discontinuities, wheel flatness, etc. The standard EN 50125-3 [37] for signaling application trackside distinguishes between positions on the rail, on sleepers, on ballast and outside the track but near to it (between 1 and 3 m); the acceleration values in the three coordinates are maximal on the rail and sleeper and then reduce, so it is highly advisable to locate all sensors at a distance of 1 m from the track and benefit from 2.3 m/s^2^ of acceleration along each axis. For shocks, there is a similar classification, and the last category indicates 20 m/s^2^ of acceleration and a duration of 11 ms.
  - Impressed Potential (IP) and pollution degree. IP 67 is recommended, not only against heavy rain but also fine dust in many countries; it is advisable to install the sensors inside a wayside small cabinet that ensures the necessary degree of protection.
  - Solar radiation and temperature. Housing inside the cabinet prevents issues due to solar radiation and UV; temperature, instead, is a major concern in railways and metros, with a wide range of values between night and day and through seasons. The standard EN 50125-3 [38] indicates temperature ranges inside cubicles of −25 °C to +70 °C for the climatic class T1 (temperate areas), although for cold areas, the minimum temperature may be as low as −55 °C.
  - Overvoltage. Overvoltages may occur for trackside devices as the consequence of a short circuit, of lightning, but also induction on cables. Section 8.2.2.2 of the EN 50124-1 [39] prescribes a rated impulse voltage of 3.1 kV for basic insulation (equivalent to a power-frequency test voltage of 1.44 kVrms). As for induction, which is relevant for a.c. systems, the standard (at its section 8.2.3) indicates that a permanent voltage of up to 250 Vrms between conductors and earth must be taken into account. It is briefly observed that induced voltage during short circuit in a.c. systems may be much larger, in the order of 1 kV [40].
2. Metrological performance:
  - Amplitude accuracy. The reliability of the SCMS output does not require extremely demanding accuracy for the measurement of the physical quantities; the standard accuracy for the measurement of track quantities is usually agreed around 1%, although the output of the measurement function even for simple setups is exposed to several non-idealities, as demonstrated in [19,20] for track insulation and insulating rail joint efficiency.
  - Offset. Focusing on the measurement of dc quantities, besides amplitude accuracy, offset is quite relevant, especially because it is not much smaller than the current values measured most of the time. Ideally, a zero offset device is preferable, or one with a very stable offset with respect to ageing and temperature, as we see in Table 1. Hall-effect probes, in general, perform less than shunts in this respect.
  - Temperature dependency. The gain of the probe (which directly influences the amplitude accuracy) has, in general, a non-negligible temperature dependency, but for dc readings, the offset is the other parameter that has, in general, a significant dependency on temperature and is particularly relevant when measuring normal small leakage of a healthy system, with output very close to the typical offset values.
  - Non-linearity. Marginally relevant for ac systems (where, however, the attention is on the fundamental rms and the error introduced by possible non-linearity is well tolerated), but not significant in case of dc quantities.

As shown in Table 1, the performance of a customized, isolated shunt resistor is superior in terms of both sensitivity and uncertainty. It is observed that the 10 A scale is not really necessary, provided that the measuring system in the 1 A scale can operate in saturation (as it is, indicating a clear out-of-scale situation) and the shunt is suitably thermally sized (at 10 A, the 1 A 50 mΩ shunt dissipates 5 W, which is within its nominal power). Electrical insulation is also much higher for the shunt solution, even exceeding the target value.

#### 3.1.3. Track Current and Track Leakage Measurement

Ideally, measuring track current at some locations in between TPSs, one knows, by difference, the amount of current leaving the track in a given section. However, rail current measurement is not trivial, as special sensors located in protected portions of space around the rail (not interfering with passing wheels) should be used, and such sensors are usually custom-made and not commercial products [42]. Otherwise, the few bonding cables around rail mechanical joints may be measured with a clamp-on probe. This approach has a basic problem of scale and sensitivity mismatching; the return current may be as large as several kA and the target quantity (the stray current leakage of a track segment) is estimated as *I_tl_P_*_1*-P*2_ = *I_tr_*(*P*_1_) − *I_tr_*(*P*_2_) with positions *P*_1_ and *P*_2_ along the rail at a conveniently large distance. Increasing this distance increases the expected stray current and improves uncertainty but loses spatial resolution, necessary for investigation and troubleshooting of local defects. By all means, *I_tl_* is about three orders of magnitude smaller than *I_tr_* and uncertainty is heavily affected. Uncompensated offsets in the *I_tr_* measurement, which may be disregarded considering the total return current intensity, come up as relevant when evaluating *I_tl_*. Offset can be estimated in time intervals without traction current flow, provided that temperature information is also available.

#### 3.1.4. Impressed Potential (IP) and Corrosion Measurements

IP measurements are at the core of the problem of assessing the potential shift of metal in an electrolyte medium (mostly rebars in concrete) against suitable limits or reference values. The electrochemical corrosion mechanisms are complex in that the effect on the read potential of various ionic species, even in small concentrations of a fraction of %, is different, and not all species cause or are relevant to corrosion. The difference in potential between the metal and the electrolyte indicates the chance of corrosion, against some reference values that necessitate the support of a minimum of interpretation for the environmental conditions and the said ionic species that may alter the potential distribution. Measurement methods and reference values to assess corrosion are considered in this order in the rest of this section.

##### Electrochemical Half-Cells

Measurements are carried out with a range of probes (called electrochemical half-cells or electrodes) that establish a good electric contact with the electrolyte (such as the concrete or the soil); they may be positioned once the transportation system is complete and already operating, e.g., during site investigations or as additional inputs to the SCMS, or at the time of construction, fastening them to reinforcement before pouring concrete. There is a range of degrees of interaction with the structure and environment, depending on the type of probe and installation; the most critical aspects are the temperature (with a double effect on electrode potential—direct, as the potential depends linearly on temperature, and indirect, in that the salt concentration of saturated electrolyte increases at higher temperatures), the solution concentration and pH, if interaction with concrete is accounted for, and possible contamination [43].

The following electrochemical electrodes (or probes) are usually applied:

- Saturated Cu/CuSO_4_ electrodes (called CSE, copper sulfate electrode) with humid sponge or porous ceramic or terracotta can be positioned on a flat surface, with care to provide sufficient wetting of the surface for the duration of the measurements, with some variability when water (or low-grade CuSO_4_ solution) is added at the interface to replace the evaporated one. They may be easily positioned in soil for long-term use. A special mix of bentonite, gypsum and a small amount of salt is used for very dry soils to preserve the necessary humidity for conduction, with minimum impact on electrochemical potential. In general, the CSE is quite insensitive to contamination from nitrite and sulfite, but chloride, even in small percentages, may definitely compromise electrode operation, which has an expected life of about 5 years in usual conditions. Copper passivation is the second weak point of this electrode. The cell potential at room temperature (20 °C) is taken as 316 mV with respect to the standard hydrogen electrode (SHE); the more accurate formula reported in [44] confirms a value of 316.8 mV (*E_CSE_* [mV] = (0.17 ± 0.01) *T* + (313.4 ± 0.04)). The temperature coefficient is usually taken as +0.9 mV/°C (with [44] showing 0.83 mV/°C with linear interpolation of measured values); although, from the results, a slight parabolic dependency seems to take place, as confirmed by [44], Figure 5. The dependency of CuSO_4_ density on temperature is well documented and stable: 1.17 g/mL @ 10 °C, 1.19 g/mL @ 15 °C, 1.21 g/mL @ 25 °C, 1.25 g/mL @ 35 °C and 1.28 g/mL @ 45 °C, as per [44], 1% larger up to 25 °C in [45].
- Saturated Ag/AgCl electrodes are used in case water is present, such as to measure corrosion of water-filled tanks and pipes. In this case, the shape of the electrode is such to be screwed in a threaded hole in the tank or pipe wall. For other uses, a special mortar is pre-cast around the electrode and sold ready for installation during construction. The temperature coefficient of the cell potential is −0.13 mV/°C; that at room temperature (20 °C) is taken as 250 mV vs. SHE in sea water but drops to about 210 mV for an electrolyte of an almost saturated solution of KCl (4 Mo/L). The operating life of the electrode depends on the amount of Ag that is lost in the reaction; being a precious metal, the objective is minimizing the employed quantity, but about 1 g of Ag allows an operating life of more than 30 years with an average exchanged current of 1 µA. As a marginal note, it is observed that the presence of chloride in concrete is what is to be avoided by all means as the most responsible for corrosion; however, this type of electrode, sealed and buffered by electrolyte and porous interfaces, is reported to be used sometimes in concrete.
- MnO_2_ electrodes are built with an electrolyte buffering the MnO_2_ from the outer mortar cap which interfaces with the rest of the concrete; the electrolyte is chosen with a pH so that it is almost the same as the one characterizing concrete, stabilizing its potential, which is highly dependent on pH [46]. Some manufacturers indicate that slight changes in the rest potential may occur after installation depending on concrete water pH. MnO_2_ electrodes are quite stable with temperature when housed in concrete or mortar. The electrode polarization is negligible provided that low current intensity is exchanged with the medium (0.1 µA or less); the voltage drop at a higher current indicates an internal resistance smaller than 1 kΩ, and old electrodes with slightly degraded concrete interface may almost double this value. Suitable minimum reading impedance at 1 V potential shift is, thus, 10 MΩ. Above this threshold, the electrode starts polarizing, with a variable equivalent internal resistance of some mV/μA at values of 1 to some µA. Care must be given to the duration of measurements; in the present case, for continuous monitoring, although at a low sampling rate, the requirement on the input impedance must be further increased, because it is the transferred charge that matters in deciding the polarization of the electrode. A 0.1–1 GΩ input impedance is, thus, much more preferred for a long operating life. The potential at room temperature is +414 mV vs. SHE or +173 mV vs. saturated calomel electrode. A statistical distribution of such potential is shown in [46], where the average value is about 173 mV and the spread is 11 mV between the minimum and maximum values of 172 samples. The electrode is best manufactured with a mortar cover (or buffer disc) that separates the internal electrolyte, prevents significant leakage and guarantees the optimal adhesion of successively poured concrete to the electrode active surface.
- The graphite/cement electrodes proposed in [47] show larger reading current without polarization issues, and for the lower tested graphite content, a behavior very similar to the Cu/CuSO_4_ electrode when immersed in concrete, with an almost constant potential shift of about 100 mV over the 4 months of reported tests. This type of electrode shares the same good properties of pure metal electrodes; negligible polarization up to some µA and good stability vs. Cl concentration. As a remarkable feature, the construction is quite easy and cheap materials are used.
- The zinc electrode has a negative potential (−0.77 V vs. SHE) and has several advantages—it does not suffer polarization, being a pure metal (so the current flow during reading is not an issue), and can be regenerated by applying a voltage that lets flow some mA/cm^2^ of anodic current for a moderate amount of time, which depends on the time interval between regenerations (let us say 1 h every 6 months, provided that the interval between regenerations is not too long, 6 months being already an upper bound). In general, it is used for measurements in sea water.
- Other pure metals may be used, such as *steel*, *black steel* and *stainless steel*, not only for potential measurements but also for concomitant measurement of polarization resistance as a direct measurement of corrosion, being such an electrode of almost the same material as reinforcement steel. The current passing through the electrode with an accurately known contact surface with concrete gives indication of the corrosion rate (see below in this section).

Temperature, in general, is the first cause of drift of electrode potential but can be, to a large extent, compensated for, in some electrode types, if the local temperature is measured with an additional sensing element. It is also remarked that the temperature inside concrete is likely to vary less than the surface temperature and, in particular, within tunnels. It is also observed that corrosion at low temperatures may be ruled out, with some cases still reported at a few degrees above 0 °C but not below.

##### Voltage Measurement Principles

As seen, measuring the potential shift requires matching the large source resistance (given by the electrolyte resistance and the polarization resistance of the electrodes) with an even larger instrument input resistance that reduces the absorbed current to comfortable values. Regarding the input impedance of the measuring instrument, the ASTM C876 [34] standard requires 10 to 200 MΩ, requesting a verification of the stability between successive readings while increasing the input impedance value (looking for stabilization of the voltage reading, meaning that undue voltage drop across the source resistance is minimized). For extreme cases, a galvanometer with some GΩ input impedance should be used, or a different technique employing a potentiometric voltmeter should be adopted. The latter is based on adjusting the internal reference voltage to compensate the unknown source voltage under measurement until the two are balanced and no current flow is observed (the reading of the internal voltage at this moment is accurate, as no voltage drop due to flowing current occurs).

A suitable common mode rejection ratio (CMRR) is also necessary, since IP values (between 100 mV and about 1 V) must be measured under a significant induced noise across connecting wires, caused by power cables and traction circuit. A minimum CMRR at mains frequency is 80 dB, by which a 100-V induced voltage translates into a 10-mV superposed ac noise, which agrees with the resolution required by the ASTM C876 standard and the typical spread and uncertainty that we have seen for the electrode dc potential.

The way the electric contact is formed is another source of instability and this is particularly troublesome for measurements on the finished system. For measurements on an accessible flat concrete surface, practical issues should be considered: cleaning and uniform and adequate wetting of the surface, and inhomogeneity of concrete in the outer layers. When, instead, the electrode must be inserted in a concrete wall, the drilled large-bore hole that hosts the electrode must then be restored with a concrete/mortar of the same type; the excess humidity must then have time to dry out and return to pristine conditions. Direct connection to a sufficiently clean rebar surface is usually achieved by means of a self-threading screw in a small, drilled hole (at the same time as the large-bore hole), automatically avoiding the issue of oxide at the rebar surface. For pipes and reservoirs, if they are not directly accessible for reinforcement, the reading is done as in the EN 13509, using half cells above the pipe and at some distance away (usually >10 m), exploiting the possibility of canceling the IR drop effects by extrapolation to an ideal off condition [48]; this technique, unfortunately, seems hardly applicable to measurements on concrete structures within the right-of-way.

##### Impressed Potential Limit Values and Indicators of Corrosion

Regarding the use of limit values to indicate the chance and/or amount of corrosion, reference can be made to impressed potential values and to polarization resistance/corrosion current values.

IP limit values can be found in the EN 50162 [25], in ASTM standards and in common practice, leading to a consolidated practice as shown in the following (values are reported with respect to CSE):

- Cases with IP ≤ −200 mV are reported as compliant or described as with a 90% or 95% probability of absence of relevant corrosion;
- When −200 mV < IP ≤ −350 mV, there is no clear behavior, although the EN 50162 already raises a warning which, however, depends on the resistivity of the medium;
- For IP < −350 mV, the chance of corrosion is 90% or 95% but also depends on the chemical species and concrete porosity.

This scale, in fact, does not explain some situations of significantly negative IP values (e.g., beyond −0.5 V and towards −1 V) that may not imply a critical corrosion scenario; the explanation given in [49] regards the oxygen concentration at the rebar–concrete interface, which is more likely to occur in the presence of a thick, dense concrete covering layer, obstructing gas passage. Conversely, in the presence of carbonation (reaction with the carbon dioxide in the atmosphere) or chloride ion concentration, negative potentials will imply steel corrosion. It is worth commenting that for carbonation, the negative potential shift may be not as remarkable as for chloride ion.

Besides potential measurements, the status of corrosion within concrete may be assessed by measuring the polarization resistance *R_p_* by the volt-amperometric method: a small voltage *e*(*t*) is applied on top of the existing corrosion potential between a rebar and the concrete [50]; the resulting current *i*(*t*) is measured and expressed as current density *j*(*t*), knowing the cross-section of the exposed area [51]. Signal dynamics must be small so as not to perturb the chemical reactions, so 10 to 30 mV maximum should be used and the rate of change of the signal should be low enough to be compatible with the mobility of the ionic species. The corrosion current flowing in the measured area is obtained from *R_p_* [Ω/cm^2^] by considering the active corroding area, as in the Stern–Geary equation.

(3) $$R_{p}={\left.\frac{\partial e}{\partial i}\right|}_{E^{\prime }\to 0}\;\;\;j=10^{6}\frac{B}{R_{p}}\;\;\;B=\frac{\beta_{a}\beta_{c}}{2.303(\beta_{a}+\beta_{c})}$$

where β*_a_* and β*_c_* are the Tefel slopes for the anodic and cathodic processes, respectively; *B* is expressed in V and the 10^6^ factor adjusts for *j* expressed in µA/cm^2^.

The intensity of the corrosion current *j* already gives some information on the condition of the reinforcement steel, for which the risk of corrosion may be rated [52]:

- For *j* ≤ 0.1 µA/cm^2^, the steel corrosion risk is negligible;
- For 0.1 < *j* ≤ 0.5 µA/cm^2^, the steel corrosion risk is low;
- For 0.5 < *j* ≤ 1 µA/cm^2^, the steel corrosion risk is moderate;
- For *j* > 1 µA/cm^2^, the steel corrosion risk is high.

The corrosion current density *j* can then be converted to a more interesting parameter for the Operator, which is the amount of steel lost due to corrosion per year, expressed in terms of penetration rate *CR* or mass loss rate *MR*.

(4) $$CR=K_{1}\frac{j}{\rho_{mat}}EW\;\;\;\;MR=K_{2}jEW$$

where *CR* is expressed in [mm (yr)^−1^]; *MR* is expressed in [g (m^2^ d)^−1^]; *K*_1_ = 0.00327 [mm g (μA cm yr)^−1^]; ρ_mat_ is the material density in [g/cm^3^]; *K*_2_ = 0.008954 [g cm^2^ (μA m^2^ d)^−1^].

The equivalent weight *EW* is dimensionless, and for pure elements, it corresponds to the atomic weight divided by the number of electrons involved in the oxidation reaction (valence). For alloys, its determination is more complex because there are several pure elements, each with a mass fraction, atomic weight and valence number; therefore, *EW* will reflect an average behavior that is tabulated in the ASTM Std. G102 [51].

Construction steel may be assigned an *EW* value of about 26–27 and a density ρ_mat_ = 7.85 g/cm^3^, so that for a current density *j* = 1 µA/cm^2^, *CR* and *MR* result into the following:

*CR* = 0.01 (±0.002) mm/yr, *MR* = 0.2373 (±0.0045) g/m^2^/d (5)

### 3.2. SCMS Physical Scope: Effectiveness of Measured Quantities

One may be interested in selecting an optimized set of measured quantities, or to know the cost–benefit relationship of adding a new quantity to the set of measured data. The effectiveness of the set of measured quantities can be considered from a control system viewpoint, where the measurement of accessible quantities (output *y*) is considered for the estimate of the internal quantity or parameter *p*, namely, in the present case, the track insulation. Provided that the target quantity is observable, the estimate will be efficient if sensitivity is large, having defined sensitivity (or Bode sensitivity) as the partial derivative of the output quantity *y* (e.g., track voltage *V_te_*) with respect to the system parameter *p* (e.g., track insulation *R_te_*):

(6) $$S_{y,p}=\frac{\partial y}{\partial p}\;\;\;\;S_{V_{te},R_{te}}=\frac{\partial V_{te}}{\partial R_{te}}$$

Low values of sensitivity will unavoidably affect the quality of the estimate. Considering, as an example, the two best candidates for the estimate of the track insulation *R_te_*, the track-to-earth voltage *V_te_* and the drained stray current *I_scd_*, it is possible to assess their effectiveness first qualitatively and then quantitatively. Assuming a delta change δ*R_te_* over a given section, the resulting change δ*I_scd_* will be proportional, having indicated with η*_scd_* the SCD efficiency for that section (which is a fixed parameter that does not depend on the electrical quantities but rather on geometry and materials) and assuming that for a reduction −δ*R_te_* in track insulation, the track leakage current *I_tl_* will be proportionally larger:

(7) $$\delta I_{tl}=-I_{tl}\frac{\delta R_{te}}{R_{te}}\;\;\;\;\delta I_{scd}=-\eta_{scd}I_{tl}\frac{\delta R_{te}}{R_{te}}$$

Conversely, the change in the local track voltage is very small as δ*I_tl_* must be compared with the full-traction return current *I_tr_*.

(8) $$\frac{\delta V_{te}}{V_{te}}=\frac{\delta I_{tl}}{I_{tr}}$$

The track voltage is influenced by the track leakage current and not by the stray current (which is the leakage current not captured by the SCP); for this reason, monitoring the *V_te_* is a possible indication of just the overall leakage, not of the amount of current leaving the track bed and reaching victim structures. The stray current can be indirectly obtained from the measurement of *I_scd_* and the knowledge of the SCD efficiency η*_scd_*.

A simple system with two TPSs at 3 km distance and a train pulling 1 kA has been simulated using the distributed parameter model described in [30]; the SCD was equipped with 6x T16 rebars (16 mm diameter), with 600 mm^2^ of steel for each rail and a total approximate longitudinal resistance *R_scd_* = 0.2 Ω/km. The track had a longitudinal resistance *R_tr_* = 18 mΩ/km with an insulation level from the SCD *R_t-scd_* = 100 Ωkm, and in turn, the SCD was very well insulated from earth with *R_scd_*_,*e*_ = 1000 Ωkm. No stray current collector was used and the drained current was brought back to the TPS through the SCD itself. A 1% reduction of the track insulation was applied. The resulting track leakage *I_tl_* was proportional and resulted in a 1% increase, as expected, confirming Equation (5). The observed change δ*V_te_* was instead proportional to *R_tr_* and the observed δ*V_te_*/*V_te_* was only 0.0002% (2 × 10^−6^), confirming Equation (6). *V_te_* sensitivity was more than three orders of magnitude smaller in all system conditions; had the overall traction current been slight larger, the disproportion would have been 10,000:1.

### 3.3. SCMS Functional Scope: Data Representation and Analysis

All measured and acquired data go into a database and they are, to various extents, correlated with the operating conditions of the trains, the traffic pattern and the amount of line loading. They are also spatially correlated because a group of data refers to the same portion of line, possibly being track voltage and SCPS current of the same track section or the impressed potential of a structure insisting on that track section. Then, they have a complex medium- and long-term time behavior because they are also correlated with environmental quantities (temperature and humidity as a function of the part of the day, of the season and of meteorological conditions) but also because of system insulation ageing (whose monitoring is one of SCMSs’ objectives). In addition, maintenance events may change conditions dramatically, causing a partial “reset” of conditions and trends.

**Figure 7:**
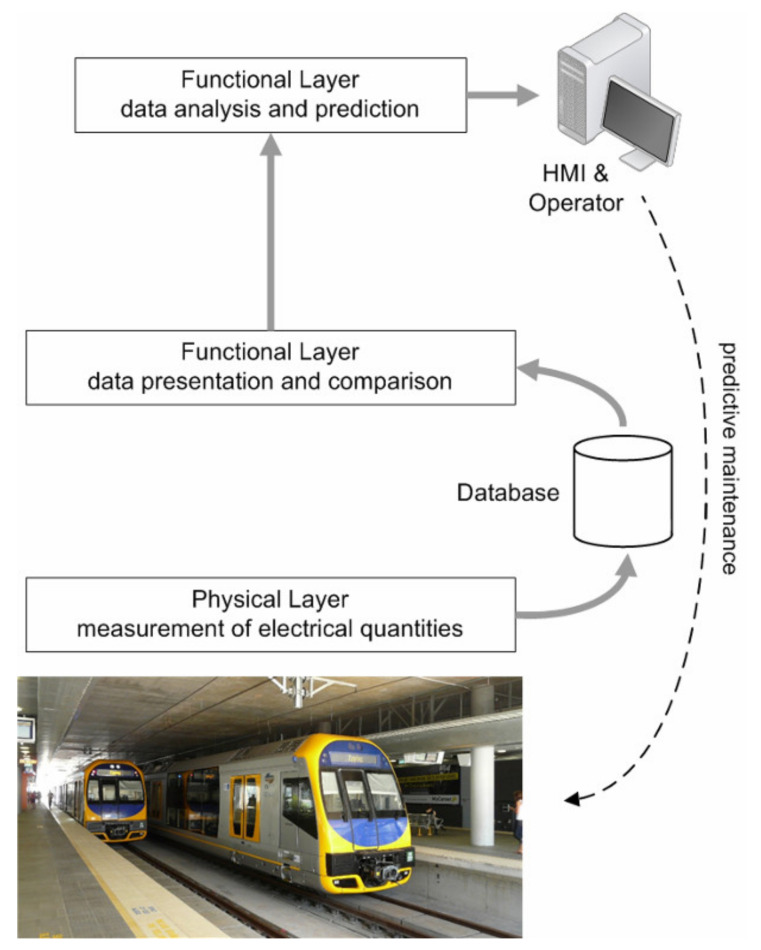
Diagram of the SCMS layers and relationships.

As shown in Figure 7, starting from a set of electrical quantities available as measured values within the SCMS database, all characterized by a satisfactory uncertainty as discussed so far, most of the added value is in the way data are processed and analyzed and then presented to the Operator (intending, with this term, both the persons dealing directly with the SCMS data and those managing and planning system maintenance and repair).

The SCMS’ objective is to provide a faithful representation of the status of track insulation and stray current impact on infrastructure and support for an Operator’s informed decision by means of data collection and presentation. The SCMS can then add a third layer on top of data measurement and collection and data presentation, which is data analysis and prediction (see Figure 7). The latter consists, in principle, in an automated and repeatable expert judgment based on available data.

Ideally, a completely effective SCMS should offer some features for data interrogation and presentation, such as:

- Data history and data search, easily implementable with a database engine, fed, however, with complete information that, besides date and time, refers to, e.g., environmental conditions and traffic conditions (data tagging).
- Data trend, which, after data extraction, presents data through time with various curve processing features, such as moving average (e.g., average over 1 day, 1 week or several weeks), median and outliers, minima and maxima and linear and higher order interpolation.
- Possibility of data comparison by exploiting data history and data search, showing results for differences and similarities between data sampled in different parts of the day (e.g., peak and off-peak hours) or week (e.g., working days and weekends) over a conveniently long time-scale (e.g., months or years). Examples of such indexes are correlation and similarity indexes (the latter is already used for validation of simulation data [23]). Similarity indexes can remove clutter due to small details, variability and spread of data and focus on the main features of the curves, although, as demonstrated in Section 2.2 and Section 3.2, the track voltage *V_te_* lacks a complete proportionality with the leakage (or stray) current and features a very low sensitivity.

A major problem to support decisions regarding the deterioration of insulation and SCP efficiency and the risk of corrosion is the definition of a baseline, where a healthy system (e.g., a new system at the end of the test and commissioning phase) is completely characterized. Besides the influence of environmental conditions on system parameters (discussed specifically for SCPS and instrumentation), system operating scenarios may be subjected to significant variability. Characterization should cover the possible changes in traffic and fleet in order to include all system parameters and consequential spread on measured quantities into the set of snapshots taken as baseline. Alternatively, different baselines may be identified for different system configurations (e.g., trains in single or double composition, peak and off-peak hours, etc.), in order to reduce the uncertainty of each baseline and improve its efficacy.

When developing a baseline, there are elements of the system and environment that have an influence on the accuracy and usefulness of such baselines; for example:

- Track insulation is influenced by the moisture content and by condensation, which may occur quickly due to environmental changes and may differ from day to day, and also, some variability between morning, afternoon and evening cannot be excluded, depending on night condensation and the amount of received sunlight (insolation).
- Driving style (for systems without automatic driving) has a significant influence on track voltage profiles; a slightly faster train will reach a local defect earlier anticipating the voltage change but will also absorb more current, resulting in a higher track voltage profile.
- Even with automatic drive and fully automatic train operation (ATO), slight delays are possible, e.g., due to high passenger density and contingency, such as doors reopening, although they should be considered a second-order influencing factor.
- Crowded or empty trains also influence acceleration and speed profiles, as well as the amount of absorbed current and, thus, the longitudinal voltage drop and the track voltage *V_te_*; as a rule of thumb, passengers’ weight in nominal occupation conditions (3–5 persons/m^2^) amounts approximately to 20–25% of a metro car weight, and passengers’ density may vary in proportion of 5:1 between off-peak and peak hours, reaching densities of up to 8–9 persons/m^2^.

As we have seen, the significance of track voltage *V_te_* for assessment of track insulation is hindered by low sensitivity values and extreme variability, with the EN 50122-1, App. B, simply indicating, as suitable, processing the calculation of minimum, maximum and average values. The spread of values is large, and using average values against a baseline is a first attempt at trend analysis. Sample dispersion, however, may compromise this type of comparison, where the observed change is in the same order of sample standard deviation. A better approach is the use of robust similarity indexes, as described in [23], where robust processing techniques are applied—for example, looking for curve features, such as peaks and anti-peaks, slopes, etc.

A further improvement, in this sense, is the preparation of some leakage reference scenarios (LRSs) by applying artificial leakage to the track for a limited time interval (see Section 4 for further details on this method), recording and processing the so-obtained track voltage curves. The comparison will then not only be against a baseline but against LRS curves that set, in this case, the amplitude of the achievable variation between classes (baseline and LRSs) to then use for testing of hypotheses, data clustering, etc.

Another aspect that is more relevant when the acquired data include the impressed potentials is the influence of external system as additional sources of stray current. The information regarding the intensity of external sources (if any) should be added to data presentation as a quantity indicating an uncertainty term of the quantification of otherwise supposedly own stray current effects. The effect of external sources should be periodically assessed when the traffic is stopped—night shifts are the first possibility, but if the external source is another transportation system or a photovoltaic plant, or even a factory, then it will also be non-operating; measurements during nights shifts are, thus, useful only to measure the natural background, such as that caused by large-scale utility or telluric currents. Instead, personnel strikes are a possibility, although uncommon, and a slight disruption to service may be considered by applying a 10 min stop to trains during off-peak hours to minimize the impact (10 min is a long enough time interval to acquire significant information).

The correlation of IP and track voltage is reported in [12] as an approach commonly used in Poland, which would alleviate the issues related to contributions external to the system. For better ETS representation, the stray current source quantity could be taken from SCPS measurements (if the SCPS is active).

## 4. Stray Current Monitoring System Verification

This section discusses the methods for the verification of SCMS performance in view of the characteristics previously considered in Section 3. Performance verification is an important step as the only guarantee that the SCMS will comply with the requirements and will detect ageing of insulation, insulation defects and other defects of the SCPS, offering an effective and reliable representation of the present status and historical data.

The methods are basically targeted at testing performance for the “physical scope” (focusing on the electrical quantities measured on the field and considering uncertainty and sensitivity) and for the “functional scope” (verifying how data are processed and presented to the Operator, besides going into the details of algorithms used for evaluation of similarity and trend and data dispersion).

### 4.1. Verification of Measurement and Acquisition of Electrical Quantities

The measurement of electrical quantities is, in general, adequately carried out with reliable hardware. As seen in Section 3.1, the critical points concern (i) the stability of offset and gain with respect to temperature and (ii) the sensitivity. Verification by test may address the first point (with site tests carried out under known different environmental conditions), but the second is better addressed by inspection of the technical characteristics.

The most straightforward approach for the verification of the measurement channels is the application of artificial signals of known accuracy (site calibration).

- For track voltage, a calibrated generator or dc voltage source may be used with a target uncertainty in the order of a fraction of %, suitable for the required SCMS metrological accuracy; as an alternative, the voltage reading may be carried out with a calibrated reference voltmeter at track for a time long enough to easily compare with the SCMS readings—4½ digital multimeters have uncertainty for the dc voltage reading in the 100 V scale better than 0.1%.
- For SCPS currents, similarly, a current should be applied either from a calibrated source or measured with a calibrated ammeter also, in this case, for a time interval long enough to be compared easily with SCMS readings; 4½ digit multimeters have uncertainty for the dc current reading in the 0.01 to 1 A scales better than 0.1%.
- For impressed potentials, most of the uncertainty is in the electrochemical probe (almost always not accessible, because it is embedded) and in the local conditions in terms of temperature, humidity and chemicals at the concrete interface (see the discussion in Section 3.1.4).

### 4.2. Verification of Usability and Completeness of Data Representation

Starting from a set of electrical quantities available as measured values within the SCMS database, most of the added value is in the way data are processed and analyzed and then presented to the Operator. The usability and completeness of this information cannot be verified by quantitative methods which would require complex mathematical models; it is preferable to assess how this information is perceived by the Operator who is the final user.

#### 4.2.1. Human–Machine Interface

A first step is the verification of the human–machine interface with concepts of ergonomics and human factors; some of the requirements for control centers of industrial process are also applicable in this context [53]. The SCMS, however, does not trigger any immediate control action, as possible control actions are switching on and off the SCPS or advising for maintenance in a specific area; both actions do not require prompt implementation as the dynamics of corrosion are slow.

Table 2 lists the requirements selected and adapted from the ISO 11064-5 [53] that are applicable in the context of SCMS, where the Operator’s task is mainly the scrutiny of data, the interrogation of the database and the request of additional service for better visualization and interpretation. The requirements are in the form of questions that may be answered on the grounds of a direct verification of the SCMS program, as well as for the most subjective points by means of a questionnaire filled by personnel interacting with the SCMS (the Operator).

Integration of high-level questions is advised, with specific examples of desirable data extraction and representation taken from past experience and knowledge of stray current and corrosion problems. Comparison with the best practice of an engineering team carrying out on-site stray current and corrosion assessment is the best approach to make the most of available data.

#### 4.2.2. Reporting

Reporting is the off-line counterpart of the HMI data presentation, having removed interactivity and dynamic information. Reports can be generated at any time and they are the only way in which information is transferred, besides the possibility of directly copying graphs and display output. Many of the questions in Table 2 for a dynamic display scenario may be transferred to a static report situation, in particular (4), (5), (11), (13), (14), (15), (16) and (18), where, in some cases, “system” is to be replaced with “report”.

### 4.3. Overall SCMS Performance Verification

**Figure 8:**
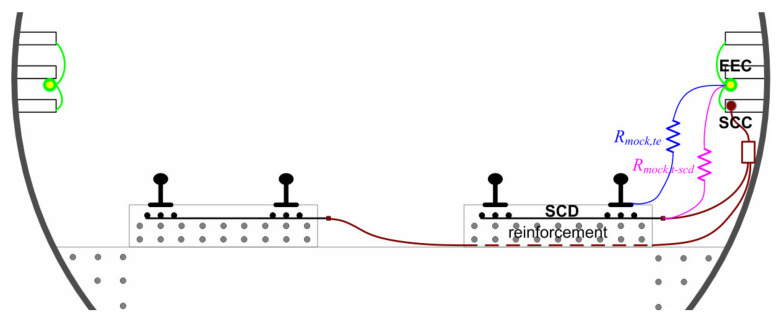
Arrangement of artificial leakage test for track alone (in blue) and track and SCD (in blue and magenta), simulating current values in line with pre-existing proportionality and the estimated SCP efficiency.

The verification of the SCMS as a whole is based on the application of known artificial leakages that must then be identified by the SCMS in due time (see Figure 8). The degrees of freedom of this verification are:

- Intensity of the leakage, which, for track insulation, may be exemplified by the value of the resistor inserted between the track and earth potential;
- Time to identification of the leakage by the SCMS, preceded by substantial deviation of the profiles of the electrical quantities (e.g., track voltage) impacted by the leakage (e.g., located in the same portion of the alignment);
- Influence of chainage (longitudinal position) where the leakage is applied and, in particular, ability to detect such leakage in front of the TPS as well as at other locations, in particular where the exchange of regenerative energy between trains is significant.

The intensity of the leakage to apply may be decided as a multiplicative factor of the existing situation (to check if and to what extent the SCMS is able to detect insulation deterioration and the beginning of a trend) or of the reference limit (contractual values or EN 50122-2 limit), in order to verify the minimum required performance, i.e., the identification of insulation failure.

As for the track, the leakage may be applied by connecting one or more resistors between the track and a conductor at the earth potential (e.g., the longitudinal earth conductor or a cable tray); the resistance value is decided as deterioration of the actual track insulation and may be applied at one point (one resistor) or distributed over a longer section (e.g., to simulate water stagnation). As an example, with a track in fairly good insulation condition of *R_te_* = 300 Ω/km, doubling of leakage over a 100-m length is achieved by paralleling the *R_te_*__100m_ = 3000 Ω with a total resistance of another 3000 Ω, resulting in a doubled leakage—this resistance may be distributed in two points separated by 100 m, using, at each point, a 6 kΩ resistor.

It must be underlined that such direct application of track leakage works for the SCMS measuring only the track voltage; the SCP quantities would not experience a corresponding increase because the leakage does not pass through the rail-to-SCD interface. An SCMS measuring all track and SCPS quantities should detect the anomaly of a conflicting behavior. This is a first check of correct operation of such an SCMS. Then the application of a realistic leakage would pass through the connection of two sets of resistors: one between the track and earth (*R_mock_*_,*t-e*_ bypassing the SCPS) and another right between the track and SCD terminals (*R_mock_*_,*t-scd*_ simulating the drained and collected current). The two added external “mock resistors” should respect the proportion of the existing mutual resistance values from track to earth and from track to SCD, so that the final *R^’^_te_* = *R_te_*//*R_mock_*_,*t-e*_ and *R*^’^*_t-scd_* = *R_t-scd_*//*R_mock_*_,*t-scd*_ keep the proportionality almost unchanged, and as a consequence, the local SCD efficiency is also preserved (assuming that the longitudinal resistance elements are much smaller than the transversal mutual elements, as it is).

As anticipated, when such artificial leakage is in place, the behavior of the SCMS must be monitored, verifying the trend of the basic and post-processed quantities and any indication of the diagnostic functions (if any). Different amounts of leakages, possibly for different time durations, will define the sensitivity of the SCMS and its diagnostic performance.

Conversely, analysis and diagnostics implemented by the SCMS should be robust against slight changes of the timetable and synchronization of trains that, indeed, affect the voltage profiles sampled per location. Such verification should be carried out by implementing the following:

- Trains during one day of service should be, e.g., randomly delayed by a small time interval, such as 10 s; this will cause a shift in the track sections where the exchange of regenerative energy occurs. Conversely, this does not change the point along the track where acceleration and braking phases begin and end because this depends on the chainage;
- Acceleration and braking phases, instead, may be varied by delaying the end and anticipating the beginning of acceleration and braking phases, respectively, at a reduced traction effort; this is achievable by applying by means of the ATO (automatic train operation) temporary speed restrictions with graded speed values.

## 5. Conclusions

Stray current, mostly for dc electrified transportation systems (ETSs), has been considered and discussed from the general viewpoint of how to experimentally assess its intensity and increase with system aging, as well as its impact on structures and installations. To this end, it is necessary to first characterize the track voltage, the resulting stray current and the necessary provisions that are part of the stray current protection, and to clarify not only the typical behavior of the involved electrical quantities but also the underlying considerations and approaches to the design. Stray current protection is implemented, besides the electrical insulation of the track, by devising a stray current protection system composed of capturing and draining sections (rebars or metallic mesh specifically located beneath the rails) joined to a collection system, which brings most of the leaking current back to the traction power station.

The impact on victim structures along the alignment caused by the track leakage straying away from the SCPS can be assessed by the assessment of the status of corrosion and the impressed potential caused by the stray current.

Stray current monitoring systems can, in principle, collect all these quantities and present them to the Operator to provide information on the ETS health status for (i) track insulation, (ii) SCPS efficiency and (iii) exposure to corrosion of reinforcement.

The measurement of such quantities has been evaluated not only for purely metrological characteristics, such as uncertainty, but also for environmental conditions and the feasibility of a specific quantity as an indicator for the previous three health status points.

The outcome is that the measurement of the SCPS quantities is more valuable to the assessment of track insulation than track voltage alone, also demonstrated by means of sensitivity functions; current measurements are preferably carried out using an isolated shunt, because commercial isolated probes have significant offset and temperature dependency, for the price range of such application.

Impressed potentials and other quantities characterizing corrosion (such as polarization resistance *R_p_* and corrosion current for the reinforcement–concrete interface) are best measured by a specialized system to then integrate into the SCMS. Such measuring systems feature a range of usable electrochemical electrodes, each with its own characteristics of accuracy, stability, immunity to pH changes and contamination, for which an expert’s judgment is advisable, although this work has discussed the pros and cons of the mostly used and available ones. Preference is oriented to pure metal electrodes, suitably sealed Ag/AgCl electrodes, MnO_2_ electrodes for low polarization current and a proposed graphite electrode. The characteristics of the system, then, are very large input impedance and the possibility of actively driving an excitation signal for *R_p_* determination.

The collection of measured data for the selected relevant quantities must then be followed by data presentation and comparison with suitable limits. Depending on the inspected quantities, criteria may be more or less defined; for track voltage, variable operating conditions and an intrinsic low sensitivity hinder its full utilization as a stray current indicator; leakage current and SCPS quantities are a more reliable, direct indication; impressed potentials are more exposed to variability and necessitate some interpretation for the influence of environmental and boundary conditions (temperature, ionic species, contamination, etc.), although they are the best choice for corrosion assessment.

An SCMS should collect, at best, all this information and present it to the Operator using customizable templates for data tagging, database interrogation and extraction, in order to make processed data understandable and reusable to support informed decisions regarding corrective actions and maintenance.

## Figures and Tables

**Table 1:** Characteristics of two measuring systems for SCPS currents.

Characteristic	Target	Method “PR”	Method “SH”
Main components	—	LEM PCM 10-P 100 Ω load res.	Ohmite PCS2512 Texas Instr. AMC1302
Current range	±1 A (SCD) ±10 A (SCC)	±20 A	±1 A (50 mΩ sh.) ±10 A (5 mΩ sh.)
Sensitivity ^(1)^	≤0.1 mA	— ^(3)^	3.4 µA 34 µA
Offset	—	167 mA	0.1 mV (2 mA) 0.1 mV (20 mA)
Gain temp. coeff. *k_G_*	≤200 ppm/°C	±500 ppm/°C	√(50^2^ + 50^2^) = 70 ppm/°C
Offset temp. coeff. *k_O_*	≤200 ppm/°C	±0.03 mA/°C	0.8 µV/°C (0.016 mA/°C) 0.8 µV/°C (0.16 mA/°C)
Overall uncertainty ^(2)^	≤2%	12.7% ^(4)^	0.87% 8.0%
Voltage insulation level	1.5 kVrms	4.9 kVrms ^(5)^	5 kVrms (1 min)
Common Mode Rejection Ratio	80 dB	—	100 dB
Output voltage @ 1A	>50 mV	80 mV	205 mV

^(1)^ Sensitivity estimated from noise specification using 1 Hz bandwidth (compatible with 1–2 Hz sampling). ^(2)^ Uncertainty estimated as standard deviation (coverage factor *k* = 1) for measured current of 100 mA, residual error after offset compensation of 2% and temperature excursion ΔT = 50 °C). ^(3)^ Output noise not specified. ^(4)^ Uncertainty mainly influenced by the offset temperature coefficient. ^(5)^ The “rated insulation voltage 50 Vrms” statement in probe datasheet [35] is ambiguous, especially if linked to the details of overvoltage category III and pollution degree 2. Voltage insulation level estimated from declared clearance, as per Table K.11 of IEC 61010-1 [41].

**Table 2:** Selected and adapted ISO 11064-5 requirements for SCMS verification.

	Selected and Adapted ISO 11064-5 Requirements
(1)	Are all functions required to cope with each situation available to the operator within reasonable time?
(2)	Is the system interruptible within 2 s by operator inputs, even when busy? Can automated functions that have no effect on the main process be stopped?
(3)	Does the operator get the information required in a timely and satisfactory way?
(4)	Does the operator have a permanent overview of the current status of the system?
(5)	Is all the information presented relevant to the task? Can irrelevant information be hidden efficiently, e.g., by category, tagging, etc.?
(6)	Is the required exchange of information during shift changes minimized by the system?
(7)	Are recurrent tasks executed by easily repeatable sequences? E.g., selection of quantities, display by chainage and time, standard processing functions.
(8)	Are infrequently used tasks self-explanatory or supported by help information?
(9)	For skilled users, are shortcuts allowed?
(10)	Is “help” support easily available?
(11)	Is the amount of information to be acquired by the operator appropriate?
(12)	Are the displayed events prioritized according to the urgency of the matter? Are the levels of attention easily distinguishable?
(13)	Is the information organized in a way that is easily recognizable and understandable by the operator?
(14)	Is the operator provided with an overview of the system at all times?
(15)	Is the operator able to tag and annotate the received data for later retrieval and analysis? Is this information also in the output report?
(16)	Is every object uniquely and unambiguously identifiable?
(17)	Is interaction with the system based on simple, easily understood concepts, with minimum set of rules?
(18)	Is the navigation through the system simple and obvious?
(19)	Can the system be used by an operator without the use of a written manual?

## Nomenclature

ATO	Automatic Train Operation	MR	Corrosion mass loss rate
CR	Corrosion Penetration rate	OVPD	Overvoltage Protecting Device
CSE	Copper Sulfate Electrode	R_mock,t-scd_	Added resistance between track and SCD
EEC	Earth equipotential conductor	R_mock,te_	Added resistance between track and earth
δV_te_	Change of track-to-earth voltage	R_p_	Polarization resistance
E_scc_	Efficiency of SCC	r_scd_	Per-unit-length resistance of SCD section
E_scd,k_	Efficiency of *k*-th SCD section	R_scd_	Resistance of SCD section
E_scp_	Overall efficiency of SCPS	*R_scd_*__200m_	Resistance of SCD section 200 m long
ETSs	Electrified Transportation System	r_scc_	Per-unit-length resistance of SCC
E_scp_	Overall efficiency of SCPS	R_te_	Track resistance-to-earth
ETSs	Electrified Transportation System	R_tr_	Track resistance
HMI	Human-Machine Interface	SC	Stray Current
I	Current pulled by the train	SCC	Stray Current Collector
I_scc_	Current in SCC	SCD	Stray Current Drainage
I_scd_	Current in SCD section	SCMS	Stray Current Monitoring System
I_tl_	Track leakage current	SCP	Stray Current Protection
I_tl,k_	Track leakage current in *k*-th section	SCPS	Stray Current Protection System
I_tl_P1-P2_	Track leakage current betw. points *P*_1_ and *P*_2_	S_scc_	Cross section of SCC
I_tr_	Track current	S_scd_	Cross section of SCD
I_tr,k_	Track current in *k*-th section	SHE	Standard Hydrogen Electrode
j	Corrosion current density	TPS	Traction Power Station
L	Train position with respect to leftmost TPS	VLD	Voltage Limiting Device
LRS	Leakage Reference Scenario	V_te_	Track-to-earth voltage
L_TPS_	Separation distance between TPSs		
